# Single-cell analysis for identification of T-cell clonotypes associated with IgG4 production of autoimmune pancreatitis

**DOI:** 10.1093/gastro/goad071

**Published:** 2023-12-08

**Authors:** Kensuke Shibata, Nao Fujimori, Takamasa Oono, Daisuke Motooka, Daisuke Okuzaki, Koh-Hei Sonoda, Yoshihiro Ogawa, Sho Yamasaki

**Affiliations:** Department of Microbiology and Immunology, Graduate School of Medicine, Yamaguchi University, Ube, Japan; Department of Ophthalmology, Graduate School of Medical Sciences, Kyushu University, Fukuoka, Japan; Department of Molecular Immunology, Research Institute for Microbial Diseases, Osaka University, Suita, Japan; Department of Medicine and Bioregulatory Science, Graduate School of Medical Sciences, Kyushu University, Fukuoka, Japan; Department of Medicine and Bioregulatory Science, Graduate School of Medical Sciences, Kyushu University, Fukuoka, Japan; Single Cell Genomics, Human Immunology, World Premier International Research Center Initiative Immunology Frontier Research Center, Osaka University, Suita, Japan; Genome Information Research Center, Research Institute for Microbial Diseases, Osaka University, Suita, Japan; Single Cell Genomics, Human Immunology, World Premier International Research Center Initiative Immunology Frontier Research Center, Osaka University, Suita, Japan; Genome Information Research Center, Research Institute for Microbial Diseases, Osaka University, Suita, Japan; Department of Ophthalmology, Graduate School of Medical Sciences, Kyushu University, Fukuoka, Japan; Department of Medicine and Bioregulatory Science, Graduate School of Medical Sciences, Kyushu University, Fukuoka, Japan; Department of Molecular Immunology, Research Institute for Microbial Diseases, Osaka University, Suita, Japan; Laboratory of Molecular Immunology, Immunology Frontier Research Center, Osaka University, Suita, Japan; Division of Molecular Immunology, Medical Mycology Research Center, Chiba University, Chiba, Japan

## Introduction

Autoimmune pancreatitis (AIP) is one of the recently established immunoglobulin G4-related diseases (IgG4-RD) [1]. The detailed pathogenic mechanisms have been an intensive research area for prophylactic and therapeutic purposes because aberrant immune activation and tissue fibrosis in AIP are the major factors that worsen the disease outcomes in these patients.

AIP is characterized by abundant infiltration of IgG4-positive plasma cells, CD4^+^ T cells, and eosinophils into the fibrotic lesions of pancreatic ducts [1]. B-cell depletion is effective treatment for patients with IgG4-RD; humoral immunity has received particular attention for its pathogenic roles [1]. Previous studies identified B cell autoantigens such as carbonic anhydrase, plasminogen binding protein, lactoferrin, pancreatic secretory trypsin inhibitor, amylase alpha-2A, trypsinogen, and annexin A11 in patients with AIP [1], indicating the presence of pathogenic B cells. These pathogenic B cells undergo maturation processes of isotype switching, somatic hypermutation, and high-affinity B-cell selection in the germinal center where the interaction with follicular helper T (T_FH_) cells plays an important role [2]. The CD4^+^ CXCR5^+^ T_FH_ cells were detected in the peripheral blood and their frequencies were increased in patients with IgG4-RD [3]. Another study showed CD4^+^ SLAM7^+^ T cells with cytotoxic activity that clonally expanded and were associated with clinical IgG4-RD symptoms [4]. It has been reported that, in a recently developed mouse model with a similar disease entity of AIP, clonally expanded *T cell receptor alpha joining* (*Traj*)*33^+^* T cells with a type 2 helper T (T_H_2)-like phenotype contributed to autoantibody productions [5].

The present study performed a single-cell RNA analysis in conjunction with T-cell receptor (scTCR-RNA-seq) analysis and identified T-cell clonotypes that correlated disease progression in patients with AIP.

## Results

**Figure 1.**
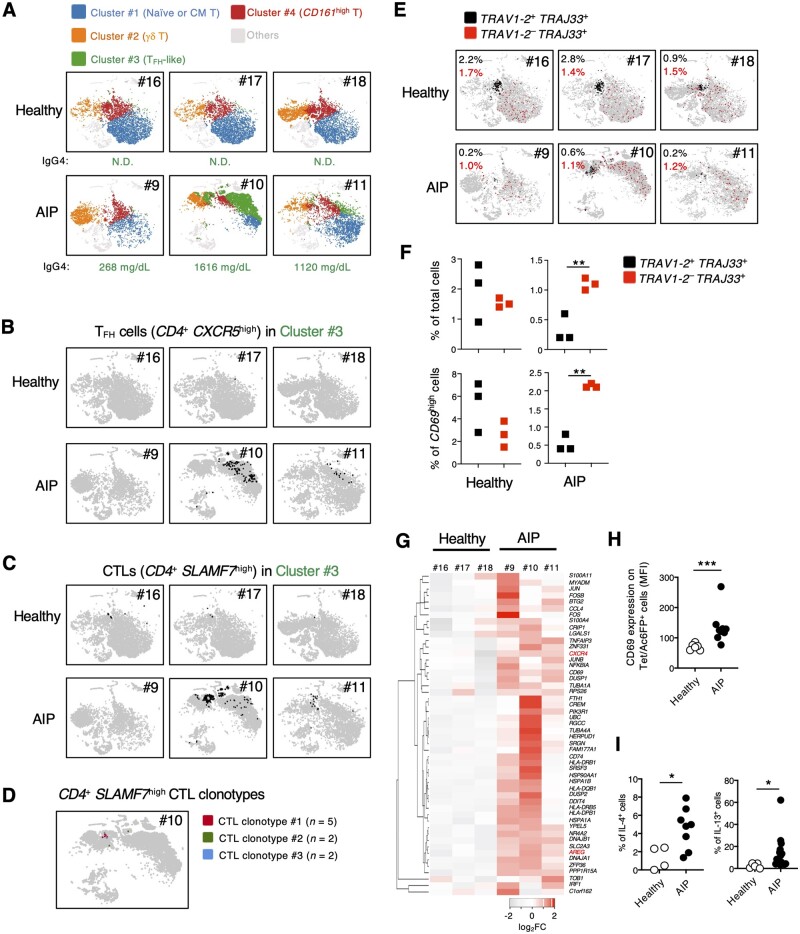
Single-cell analysis of T cells in patients with AIP. (A)–(E) tSNE plots generated by data based on single cell transcriptome and TCR analysis of the indicated individuals. (A) Cell clusters are generated by the K-means cell-clustering algorithm. “Significant gene” analysis is performed by the Loupe Browser. Colored dots indicate T-cell subsets. (B) *CD4*^+^*CXCR5*^high^ T_FH_ cells or (C) *CD4*^+^*SLAMF7*^high^ CTLs in Cluster #3 are highlighted. *CD4*^+^, *CXCR5*^high^, and *SLAMF7*^high^ populations were identified by log_2_ fold change of >0, 0.5, and 0.5, respectively. (D) Clonally expanded three *CD4*^+^*SLAMF7*^high^ CTL clonotypes in Cluster #3 are shown. Numbers in parentheses show the detected clonotype numbers in this analysis. (E) Colored dots indicate T-cell subsets. (F) Percentages of total (upper panels) or *CD69*^high^ (lower panels) cells in indicated populations calculated from the data in (E). (G) Heat-map analysis of comparative gene expressions in *TRAV1-2*^−^*TRAJ33*^+^ cells between healthy donors and patients with AIP conducted by using Heatmapper (http://www.heatmapper.ca). T_FH_ marker genes *CXCR4* and *AREG* are highlighted. The data were acquired from Figure 1A. (H) Each plot represents the MFI of CD69 expression in Ac-6-FP-tet^+^ cells of healthy donors and patients with AIP. (I) Percentages of IL-4^+^ and IL-13^+^ cells within Ac-6-FP-tet^+^ cells from healthy donors and patients with AIP after stimulation with PMA and ionomycin. (F), (H), and (I) Statistical significance was determined by using unpaired two-tailed Student’s *t*-test (*P < 0.05, **P < 0.01, ***P < 0.001). AIP = autoimmune pancreatitis, tSNE = t-distributed stochastic neighbor embedding, TCR = T-cell receptor, CM = central memory, T_FH_ = follicular helper T, CTL = cytotoxic T lymphocyte, MFI = mean fluorescence intensity, PMA = phorbol 12-myristate 13-acetate.

CD3^+^ cells were enriched from peripheral blood mononuclear cells of patients with AIP before steroid treatment and healthy donors to examine T-cell populations that were associated with clinical AIP symptoms. These CD3^+^ cells underwent scTCR-RNA-seq analysis followed by t-distributed stochastic neighbor embedding (tSNE)-based clustering. The dimension reduction analysis revealed four clusters that were present in all samples (Figure 1A). Among these clusters, we observed a clear population size reduction in Cluster #1 (blue dots) in patients with AIP (#9 and #10) as compared with healthy controls. Cluster #1 demonstrated high expressions of *LEF1*, *SELL*, *TCF7*, *CCR7*, and *ITGA6* (Supplementary Table 1), which are reportedly known as markers for naive or central memory (CM) T cells [6]. In sharp contrast, Cluster #3 was markedly enlarged in two patients with AIP (#10 and #11) despite the difference between the individuals (Figure 1A). The cluster size associated well with serum IgG4 levels (Figure 1A). Further analysis revealed high expressions of T_FH_-cell signature genes, such as *PDCD1*, *IL21*, *CXCR4*, *ICOS*, and *CXCL13*, in Cluster #3 [7] (Supplementary Table 1). Accumulations of T_FH_ cells that are identified by *CD4* and *CXCR5* expressions were observed in Cluster #3 of patients with AIP (Figure 1B, black dots). Moreover, *CD4*^+^*SLAMF7*^high^ cytotoxic T-lymphocyte (CTL) populations were present in Cluster #3 (Figure 1C, black dots) and, among them, three clonotypes with more than two cells were observed in Patient #10 (Figure 1D and Supplementary Table 2), indicating an antigen-dependent proliferation as previously proposed [4]. Compared with Clusters #1 and #3, Clusters #2 and #4 had rather smaller differences in size between patients with AIP and healthy controls. Cluster #2 specifically expressed *TRDC*, *TRGC1*, and *TRGC2*, indicating a γδ T-cell population. Cluster #4 uniquely expressed *KLRB1* (encoding *CD161*), which is a marker for mucosal-associated invariant T (MAIT) cells that recognize microbial metabolites [8] (Supplementary Table 1). These results indicate that T_FH_-like *CD4*^+^*SLAMF7*^high^ CTLs are associated with serum IgG4 production.

*TRAJ33* ^+^ T cells contain at least two types of T-cell subsets that recognize microbial metabolites. The major populations of *TRAJ33*^+^ T cells rearranged with the *TRAV1-2* segment (*Trav1* in mice) are also known as MAIT cells [8]. MAIT cells recognize vitamin B-based metabolites presented by an antigen-presenting molecule, the major histocompatibility complex class I-related molecule 1 (MR1) [8]. Another population is *TRAJ33*^+^ T cells, which are rearranged with the non-*TRAV1-2* segment and recognize unknown metabolites presented by MR1 [8]. We previously used a mouse model that shares clinical symptoms with AIP and revealed that the MR1-restricted *Trav1*^−^*Traj33*^+^ non-MAIT cell population is associated with disease symptoms [5]. MAIT cells, defined by canonical *TRAV1-2-TRAJ33* (human counterpart of mouse *Trav1–Traj33* gene segment) expression, were present (Figure 1E, black dots in upper plots) within a *CD161*^high^ αβ T-cell cluster in healthy donors (Figure 1A, red dots), while such a cluster was unclear in patients with AIP (Figure 1E, black dots in lower plots). Instead, *TRAV1-2*^−^*TRAJ33*^+^ T cells were present in patients with AIP at a comparable level to healthy donors (Figure 1E, red dots) and the frequency of these cells with the high expression level of the activation marker *CD69* was significantly higher in patients with AIP than in healthy donors (Figure 1F). The *TRAV1-2*^−^*TRAJ33*^+^ T cells from patients with AIP compared with healthy donors express *CXCR4* and *AREG* (Figure 1G), which are characteristics of recently reported type 2 pathogenic T cells [9, 10]. MR1-restricted *TRAV1-2*^−^*TRAJ33*^+^ T cells were further assessed by staining with acetyl-6-formylpterin (Ac-6-FP)-loaded hMR1 tetramers (Ac-6-FP-tet) [8]. Hence, we generated human MR1 tetramers and analysed patient samples after loading with Ac-6-FP (Supplementary Figure 1). The Ac-6-FP-tet^+^ αβ T cells expressed significantly higher CD69 levels on the cell surface in patients with AIP than in healthy donors (Figure 1H). Furthermore, the frequencies of interleukin (IL)-4^+^ and IL-13^+^ Ac-6-FP-tet^+^ T cells were significantly increased in the patients with AIP (Figure 1I). Altogether, these results indicate that *TRAV1-2*^−^*TRAJ33*^+^ T-cell frequencies that possess a pathogenic T_H_2 profile are increased in the periphery of patients with AIP.

## Discussion

Recent advances in single-cell analysis technology have enabled a more detailed and *in vivo* dynamic examination of individual immune cells. We used this technology to identify the correlation of T-cell clonotypes with clinical AIP symptoms. Help from particular T-cell subsets including T_FH_ cells and T_H_2 cells is essential for high-affinity antibody production by B cells, which are major pathogenic drivers of many autoimmune diseases, including AIP. Following the T–B interaction, T cells undergo clonal expansion upon recognition of cognate antigens presented by B cells. Therefore, identifying clonally expanded T-cell clonotypes with T_FH_- and T_H_2-like signatures will provide a valuable insight to estimate disease progression in AIP.

## Supplementary Material

goad071_Supplementary_Data
