# Supplementary material for: Single-cell analysis for identification of T-cell clonotypes associated with IgG4 production of autoimmune pancreatitis
Source: Gastroenterol Rep (Oxf). 2023 Dec 8;11:goad071. doi: 10.1093/gastro/goad071 (PMC10709539; doi:10.1093/gastro/goad071)
Supplement: goad071_Supplementary_Data [file goad071_supplementary_data.docx]

Single cell analysis for identification of T cell clonotypes associated with clinical symptoms of autoimmune pancreatitis

# Supplemental materials

**Supplementary Figure 1. Analysis of human MR1-restricted T cells by using hMR1 tetramer**

(A, B) Characterization using human (h) MR1 tetramers. 5-OP-RU or Ac-6-FP was added to the Human K43A MR1 tetramer at a concentration of 50 μg/mL. (A) A human MAIT TCR-expressing TCRα^−^β^−^ hybridoma (clone 4L4T) and its parental cell line were analyzed after gating on viable cells. (B) After staining PBMCs with hMR1 tetramers, cells were analyzed by gating on CD3^+^ cells. Numbers indicate the percentage of cells within the indicated regions.

MR1, major histocompatibility complex class I–related molecule 1; MAIT, mucosal-associated invariant T; TCR, T cell receptor; PBMC, peripheral blood mononuclear cell.

## Methods

### Human subjects

The study design and methods were approved by the Institutional Review Board of Center for Clinical and Translational Research of Kyushu University Hospital (IRB serial number: 2020-656). The methods were carried out in accordance with the approved guidelines. All patients or their relatives gave their informed consent within written treatment contract on admission and therefore prior to their inclusion in the study. Patients before steroid treatment were enrolled in this study (**Supplementary Table 3**). Mononuclear cells were prepared using BD Vacutainer CPT (Cat. No. 362761; BD Biosciences, New Jersey, USA).

### Compounds

Ac-6-FP (Cat. No. 11.418) was purchased from Schircks laboratories (Bauma, Switzerland). 5-A-RU (Cat. No. A629245) was purchased from Toronto Research Chemicals (Ontario, Canada). Methylglyoxal solution (Cat. No. M0252) was purchased from Sigma (Missouri, USA). 5-OP-RU was generated by reacting 5-A-RU with equal molar ratio of methylglyoxal. 5-OP-RU concentrations are shown under assumption that all 5-A-RU is converted to 5-OP-RU.

### Flowcytometric analysis

FITC-conjugated anti-human (h) CD3ε (UCHT1; Cat. No. 300405) mAbs, PECy7- conjugated anti-hVα7.2 (3C10; Cat. No. 351711), PerCP-Cy5.5-conjugated anti-hCD19 (HIB19; Cat. No. 982412), anti-hTCRγ/δ (B1; Cat. No. 331223) and anti-hCD14 (63D3; Cat. No. 367109) mAbs, APC-conjugated anti-hIL-4 (MP4-25D2; Cat. No. 500811) mAbs, BV421-conjugated hCD69 (FN50; Cat. No. 310929) and hIL-13 (JES10-5A2; Cat. No. 501915) mAbs were purchased from BioLegend (San Diego, USA). Dead cells were stained with 7AAD (7-amino-actinomycin D; Cat. No. 420403; BioLegend) and viable cells were analyzed by FACS Verse (BD Biosciences) or Gallios flow cytometer (Beckman Coulter, Calfornia, USA). For analyzing cytokines such as hIL-4 and hIL-13, cells were analyzed by intracellular staining method after stimulation with phorbol 12-myristate 13-acetate (PMA; Cat. No. P8139; Sigma) and ionomycin (Cat. No. I0634; Sigma) for 4 hr. Brefeldin A (Cat. No. B7651; Sigma) was added for last 3 h. The data were analyzed using FlowJo software (BD Biosciences).

### Preparation and staining of mouse and human MR1 tetramers

Human K43A MR1 tetramer was purchased from Medical &Biological Laboratories Co., LTD. Cells were stained with tetramers for 30 min on ice. After tetramer staining, cells were counterstained with anti-APC unconjugated mAb (Cat. No. APC003; BioLegend) for 20 min on ice and then surface staining with various antibodies as indicated above was performed.

### Single cell-based transcriptome and TCR analysis

Libraries for human T cells were prepared using following reagents (10X Genomics, Calfornia, USA); Chromium Single Cell 5′ Library & Gel Bead Kit, PN-1000120; Chromium Single Cell V(D)J Enrichment Kit, Human T Cell, PN-1000005; Chromium Single Cell 5’ Library Construction Kit, PN-1000020; Single Index Kit T Set A, PN- 1000213; Chromium Single Cell 5' Feature Barcode Library Kit, PN-1000080; Single Index Kit N Set A, PN-1000212. Approximately 2 × 10^4^ cells are loaded into Chromiu microfluidic chips to generate single-cell gel-bead emulsions using the Chromium controller (10X Genomics). Suspensions containing ∼16,000 cells were loaded on the instrument. RNA from each sample was subsequently reverse-transcribed in a Veriti Thermal Cycler (Thermo Fisher Scientific, Massachusetts, USA), and all subsequent steps to generate single-cell libraries were performed according to the manufacturer’s protocol, with 14 cycles used for cDNA amplification. Then ∼50 ng of cDNA was used for gene expression library amplification by 14 cycles in parallel with cDNA enrichment and library construction for T cell libraries. Fragment size of the libraries were confirmed with the Agilent 2100 Bioanalyzer (Agilent, Calfornia, USA). Libraries were sequenced on an Illumina NovaSeq 6000 as paired-end mode (read1, 28bp; read2, 91bp). The raw reads were processed by cellranger 3.1.0 (10X Genomics). Clonotype analysis was done using Loupe Cell Browsers provided by 10x Genomics (https://support.10xgenomics.com/single-cell-gene-expression/software/downloads/latest#loupetab). After identification of TCR clonotypes by the loupe V(D)J browser, transcriptome analysis of these clusters was further performed by the loupe browser.

### Data availability

scRNA-seq data have been deposited at the DNA Data Bank of Japan (DDBJ) database under the accession number DRA011320.


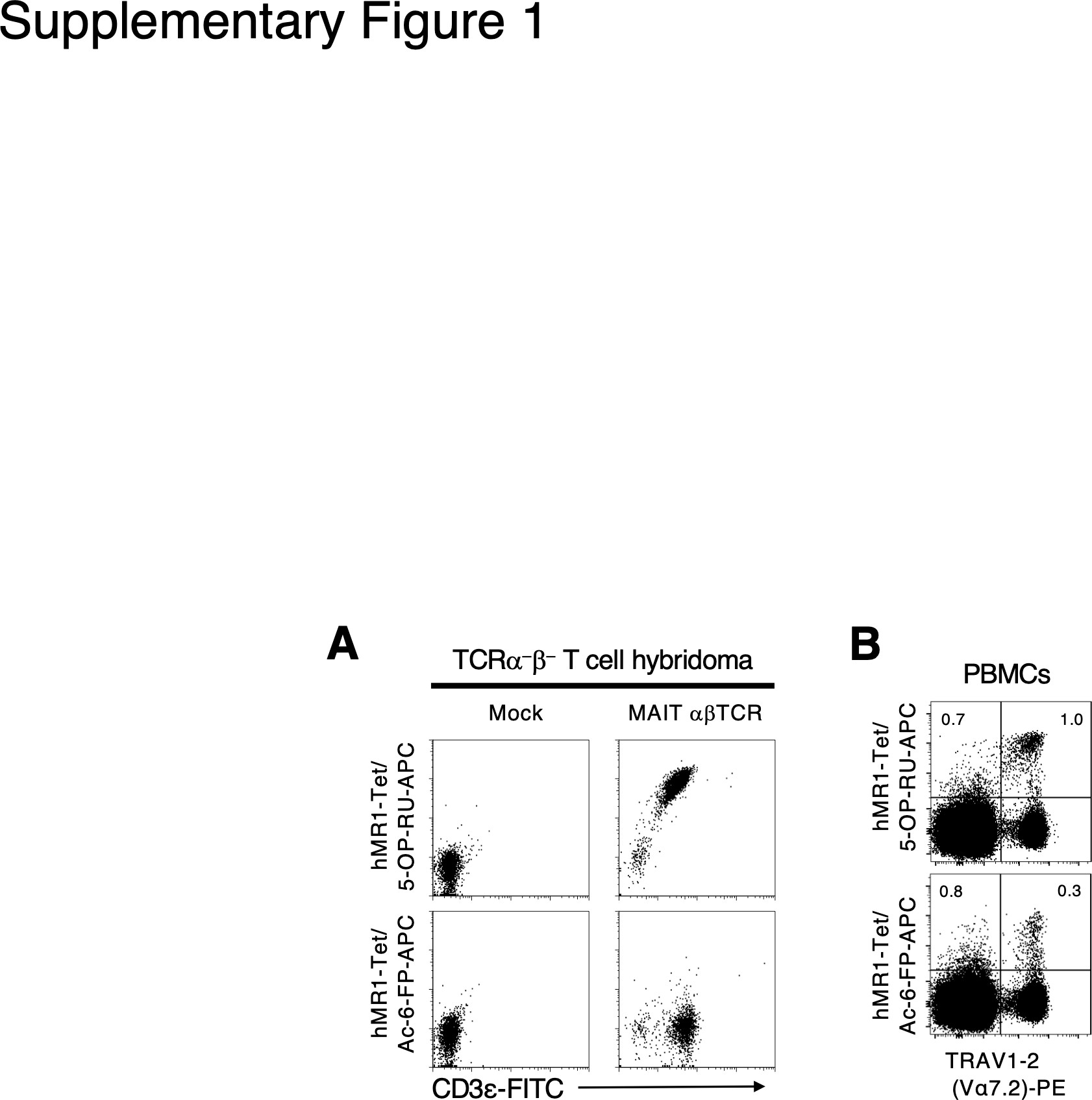


**Supplemantary Table 1**. Significantly upregulated genes in each cell cluster observed by human scRNA-seq analysis

| **Cluster 1** | | |
| --- | --- | --- |
| Gene name | Fold change (Log_2_) | *P*-value by K-means clustering method |
| *LEF1* | 2.07 | 6.44E-13 |
| *TCF7* | 1.76 | 1.63E-09 |
| *CCR7* | 1.69 | 8.32E-09 |
| *ITGA6* | 1.44 | 2.31E-06 |
| *SELL* | 0.97 | 0.002797638 |

|  | **Cluster 2** |  |
| --- | --- | --- |
| Gene name | Fold change (Log_2_) | *P*-value by K-means clustering method |
| *TRDC* | 4.30 | 7.41E-45 |
| *TRGC1* | 3.89 | 1.17E-35 |
| *TRGC2* | 2.69 | 9.09E-16 |

| **Cluster 3** | | |
| --- | --- | --- |
| Gene name | Fold change (Log_2_) | *P*-value by K-means clustering method |
| *PDCD1* | 1.58 | 1.88E-05 |
| *IL21* | 1.41 | 0.006474584 |
| *CXCL13* | 2.24 | 0.02384651 |
| *ICOS* | 0.96 | 0.028310361 |
| *CXCR4* | 0.92 | 0.03739283 |

| **Cluster 4** | | |
| --- | --- | --- |
| Gene name | Fold change (Log_2_) | *P*-value by K-means clustering method |
| *KLRB1* | 1.32 | 0.000706427 |

**Supplemantary Table 2**. Clonally expanded CTL clonotypes

| Clonotype # | TCRα | CDR3α | TCRβ | CDR3β |
| --- | --- | --- | --- | --- |
| 1 | *TRAV13-1/TRAJ11* | CAASIDSGYSTLTF | *TRBV5-6/TRBJ2-1* | CASSFTGEQFF |
| 2 | *TRAV21/TRAJ54* | CAVRPQGAQKLVF | *TRBV29-1/TRBJ2-1* | CSVSVGGPEQFF |
| 3 | *TRAV14-DV4/TRAJ34* | CAMRELSYNTDKLIF | *TRBV28/TRBJ2-7* | CASSLPDFSATYEQYF |

**Supplementary Table 3.** Patients and healthy donors enrolled in this study

| Case # | Age | Sex | IgG4 (mg/mL) | Disease onset or relapse |
| --- | --- | --- | --- | --- |
| 1 | 80 | male | 388 | Relapse |
| 2 | 65 | male | 846 | Disease onset |
| 3 | 64 | male | 229 | Relapse |
| 4 | 66 | male | 185 | Disease onset |
| 5 | 48 | male | 1047 | Disease onset |
| 6 | 72 | male | 215 | Disease onset |
| 7 | 58 | male | 395 | Disease onset |
| 8 | 61 | male | 200 | Disease onset |
| 9 | 67 | female | 268 | Disease onset |
| 10 | 80 | male | 1616 | Relapse |
| 11 | 59 | female | 1120 | Disease onset |
| 12 | 26 | male | N.D. | No |
| 13 | 28 | female | N.D. | No |
| 14 | 29 | male | N.D. | No |
| 15 | 30 | male | N.D. | No |
| 16 | 22 | female | N.D. | No |
| 17 | 23 | male | N.D. | No |
| 18 | 22 | male | N.D. | No |

IgG4, immunoglobulin G4; N.D., not determined.
